# Impact of a Narrow Coastal Bay of Bengal Sea Surface Temperature Front on an Indian Summer Monsoon Simulation

**DOI:** 10.1038/s41598-018-35735-3

**Published:** 2018-12-06

**Authors:** Dhrubajyoti Samanta, Saji N. Hameed, Dachao Jin, Vishnu Thilakan, Malay Ganai, Suryachandra A. Rao, Medha Deshpande

**Affiliations:** 1 Environmental Informatics, University of Aizu, Aizu-wakamatsu, Japan; 2 Present Address: Asian School of the Environment, Nanyang Technological University, Singapore, Singapore; 3 Present Address: Collaborative Innovation Center on Forecast and Evaluation of Meteorological Disasters, Nanjing University of Information Science and Technology, Nanjing, China; 4 Present Address: Department of Earth and Environmental Sciences, Indian Institute of Science Education and Research Bhopal, Bhopal, India; 5 Indian Institute of Tropical Meteorology, Pune, India

## Abstract

A dry bias in climatological Central Indian rainfall plagues Indian summer monsoon (ISM) simulations in multiple generations of climate models. Here, using observations and regional climate modeling, we focus on a warm coastal Bay of Bengal sea surface temperature (SST) front and its impact on Central Indian rainfall. The SST front, featuring sharp gradients as large as 0.5 °C/100 km, is colocated with a mixed layer depth (MLD) front, in a region where salinity variations are known to control MLD. Regional climate simulations coupling a regional atmospheric model with an ocean mixed layer model are performed. A simulation with observed MLD climatology reproduces SST, rainfall, and atmospheric circulation associated with ISM reasonably well; it also eliminates the dry bias over Central India significantly. Perturbing MLD structure in the simulations, we isolate the SST front’s impact on the simulated ISM climate state. This experiment offers insights into ISM climatological biases in the coupled NCEP Climate Forecast System version-2. We suggest that the warm SST front is essential to Central Indian rainfall as it helps to sustain deep and intense convection in its vicinity, which may be a source for the vortex cores seeding the monsoon low-pressure systems.

## Introduction

The Indian summer monsoon (ISM) rainfall, accounting for about 80% of the annual rainfall over the Indian subcontinent, affects the economy and livelihood of over a billion people in the region greatly^1^. Thus, understanding and predicting ISM variations assume great practical importance. Coupled General Circulation Models (CGCM) offer the best way forward for ISM prediction, because only these complex models are capable of representing the multitude of factors thought to influence ISM^2^. These factors involve a variety of timescales and dynamical mechanisms, from monsoon depressions^3^ and intraseasonal oscillation to interannual climate modes such as the Indian Ocean Dipole^4^ and El Niño Southern Oscillation^5–7^. Recent studies suggest that accurate simulation of the climatological state is necessary for skillful prediction of the ISM^8^. However, a major problem with many CGCMs is that their ISM climatological states are severely biased^9,10^. Therefore, understanding and correcting climatological biases in CGCMs provide a right step in improving ISM prediction.

In this study, we focus on the ISM rainfall bias in the National Centre for Environmental Prediction (NCEP) Climate Forecast System version-2 CGCM (hereafter CFSv2)^11^. The CFSv2 is the proposed dynamical seasonal prediction system for the Indian National Monsoon Mission (http://www.tropmet.res.in/monsoon/files/about_us.php). It is noted to have the highest forecast skill for the retrospective forecast of ISM rainfall among the models of similar class^12^, and has strong skill in simulating large-scale features including sea surface temperature (SST), winds and rainfall. This model also simulates different modes of climate variability^13–15^, as well as the monsoon intraseasonal oscillations^16^ reasonably well.

**Figure 1:**
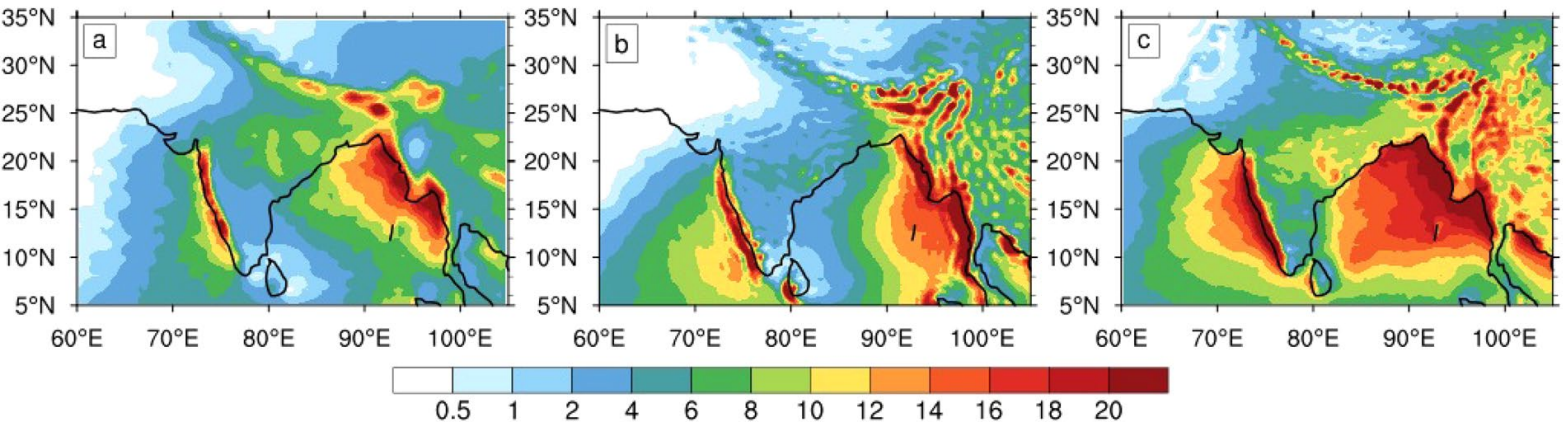
Seasonal climatology of rainfall. Rainfall climatologies (unit: mm/day) for (**a**) observations, (**b**) CFSv2, and (**c**) OML_OBS. Three major rainfall zones are seen in the observations: over the western ghats, Central India, and northern BoB. OML_OBS reproduces rainfall that closely matches the observations, although it overestimates rainfall over the oceans. The major zones of ISM rainfall are well captured by OML_OBS. Note that the dry bias of CFSv2 over Central India is not present in the OML_OBS simulation.

Yet, like most other CGCMs, a notable and serious climatological bias in CFSv2 is the dry bias^17,18^ over Central India (Figs 1b and S1a), the northern Bay of Bengal (BoB), and along the Western Ghats. Over these key zones of intense ISM rainfall (Fig. 1a), CFSv2 underestimates rainfall intensity and variations in the synoptic and longer scales. In particular, the dry bias is severe over Central India and the associated monsoon trough region; over these regions, CFSv2 underestimates rainfall to the order of 2–8 mm/day (Fig. S1a). Besides the dry bias over the land regions, the model has a wet bias (Figs 1b and S1b) over the Arabian Sea, the southern BoB, and the eastern equatorial Indian Ocean region.

Part of CFSv2’s rainfall bias is likely related to model resolution and may arise from the inadequate representation of mesoscale topographic features such as the steep slopes over the western side of the Western Ghats and Tibetan plateau^19,20^. It is also possible that one or more key physical processes relevant to ISM rainfall may not be well represented in the CFSv2 model. A recent study^16^ related the underestimation of both mean and variability of ISM rainfall in CFSv2 to deficiencies in the simulation of tropospheric temperature. Others point out to the severe biases of ocean state in the model, which manifest as SST biases. Attempts to correct SST bias in CFSv2 while somewhat improving monsoon intraseasonal oscillations and predictability of active-break spells, still fail to reduce the dry bias over Central India^21^.

**Figure 2:**
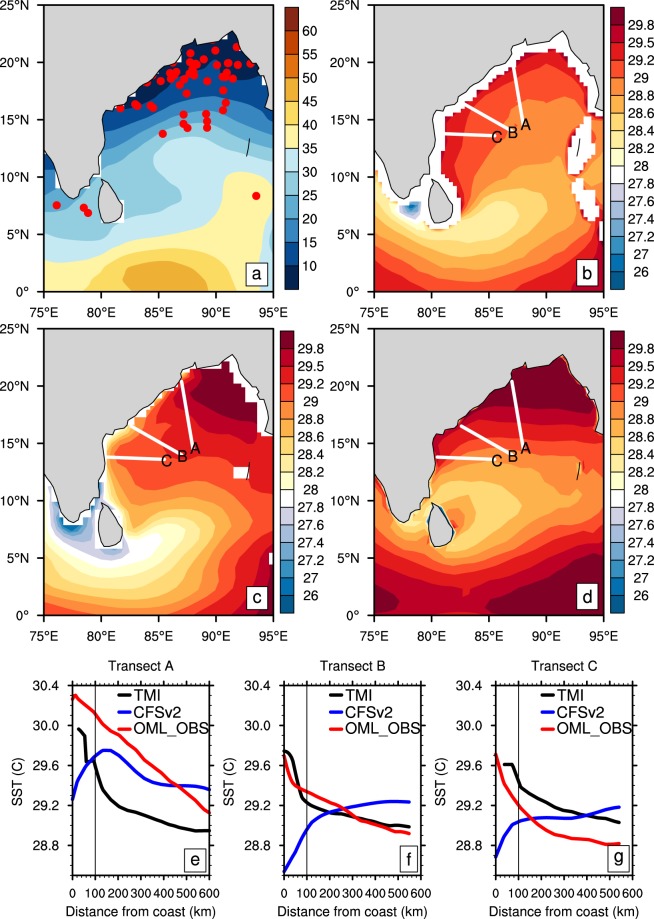
Spatial structure of seasonal climatological mixed-layer depth (MLD) and sea surface temperature (SST) over the BoB. (**a**) Observed MLD (unit: m) is overlaid with the estimated genesis locations of monsoon depressions over the period 1979–2015; climatological SST (unit: °C) for (**b**) observations, (**c**) CFSv2, and (**d**) OML_OBS. (**e**,**g**) Climatological SST along three transects (labeled A, B, C in Fig. 2b–d) that transverse the SST gradient. A sharp rise of SST within a 100 km from the coast is clear along all the three transects, with the front being the strongest near the location of transect B. The start and end location of the transects are as follows: A(20.4°N, 87.0°E to 14.5°N, 88.0°E), B(16.5°N, 82.6°E to 14.0°N, 87.0°E) and C(13.8°N, 80.5°E to 13.6°N, 85.5°E).

A large fraction of Central India rainfall where the severe dry bias persists is due to low-pressure systems (hereafter LPS; including lows and monsoon depression) propagating inland from the BoB^22–25^. The majority of this LPSs originate in the northwestern BoB, where the mixed layer depth (MLD) is very shallow (5–10 m, Fig. 2a)^26,27^. As weak winds present over this region cannot overcome the strong near-surface stratification due to low-salinity surface layer, the mixed layer remains remarkably shallow^26,28^. The shallowness of mixed layer makes the region extremely sensitive to the atmospheric heat fluxes associated with the monsoonal flow; the unusually large SST fluctuations over this region on diurnal and intraseasonal time scales^29^ reflect this characteristic of the MLD.

Since the low-salinity tongue is confined to the coastline, the MLD also exhibits strong gradients directed across the coastline, that is reflected in strong SST gradients with a similar orientation in the observations. Figure 2b shows that a very narrow coastal SST front is associated with this MLD structure over the northwestern BoB. To demonstrate the features of the SST front, we plot three transects in the northwestern BoB (labeled A, B, C in Fig. 2b–d) that transverse the SST gradient. The observed climatological SST along these transects is shown in Fig. 2e,f as the black curve. A sharp rise of SST within 100 km from the coast is clear along with all the three transects, with the front being the strongest near the location of transect B.

However, this narrow coastal SST front in the northwestern BoB is poorly simulated in CFSv2 (Fig. 2c,e–g), where not only SST is underestimated^30^, but the SST gradients normal to the coast are also reversed. Many CGCMs, including CFSv2, overestimate the contribution of local, east-coast winds, which favour upwelling (lower sea level) during ISM. This may lead to the underestimation of SST over the warm front in the CFSv2 simulations^31^.

Previous studies have shown that spatial gradients of SST are as important^27^ as the mean SST in generating deep atmospheric convection in the BoB. Further, Robinson *et al*.’ s^32^ idealized simulations suggest that deep convection in the atmosphere is particularly strong when the surface heating has a horizontal scale that can excite a resonance with respect to the propagation of internal gravity waves. The 50 km resonant scale found under realistic conditions^32^ is close to the scale of the SST front along the coastlines of the northwestern BoB. The interaction of the BoB vortex with westward vertical shear, and vortex stretching is closely associated with latent heat, and crucial for breeding and westward propagation of LPS^33,34^. Arguably, the simulation of BoB SST, in particular, the coastal SST front, is important not only for the simulation of convection over the BoB, but also for correctly simulating the LPSs that are responsible for a large fraction of the rainfall over Central India.

A recent study^35^ showed that mean SST and air-sea interaction in the Indian Ocean has a great control over LPS structure in CFSv2. Prescribing a flux-driven SSTs in the Indian Ocean (i.e. replacing cold bias by a warm bias), they further showed a better representation of LPSs and Central Indian rainfall in CFSv2. However, suppressing the coupled dynamics results in a significant drop in ISM rainfall variance^36^. Therefore, a better representation of physical processes associated with coupled ocean dynamics, especially the BoB mixing remains a challenge for a realistic simulation for LPSs, Central Indian rainfall and mean ISM climate^37^.

Motivated by the above considerations, we seek to explore how better simulation of SST over the BoB, especially the SST front near the northwestern BoB, where LPSs originate, can mitigate the dry bias observed in CFSv2. Restricted by computational resources, we did not directly modify/experiment with the CFSv2 model. Instead, we drove a regional climate model, the Weather Research and Forecast (WRF) model, with lateral boundary conditions from the CFSv2 over tropical Indian Ocean region (domain is shown in Fig. S2). We note that these regional simulations preserve the large-scale structure of the ISM simulated by CFSv2 and its temporal evolution (Fig. S3). Since the dynamics and physical processes incorporated in the regional climate model are (at large) different from those in CFSv2, there are differences between the CFSv2 and WRF simulations. However, by perturbing only the lower boundary conditions in otherwise identical regional climate run, we hope to get insights into how SST structure in the BoB affects the simulated monsoon. We conducted these perturbed runs by coupling a simple one-dimensional oceanic mixed layer model to WRF, and by varying the structure of the mixed layer between the experiments. In one experiment, which we refer to as OML_50M, the mixed layer was kept at a uniform depth of 50 m. In a parallel experiment, the OML_OBS experiment, the depth of the model’s mixed layer was set to a spatially varying observed climatological value.

## Results

Due to the realistic representation of the mixed layer structure, the OML_OBS experiment reproduces the large-scale spatial structure of climatological SST in the BoB (Fig. 2d) reasonably well. The coastal SST front along the western BoB, in particular, is captured well compared to observations (Fig. 2e,f). However, the SSTs simulated with the mixed-layer formulations (OML_OBS and OML_50M) have a warm bias, because the formulations do not include oceanic processes that remove the heat from the BoB^36^. Note that, while describing these experiments, we subtract 0.5 °C from the SST data to facilitate comparison of its structure with data from other sources. In spite of the warm bias, the OML_OBS experiment realistically captures the increase of SST towards the coastal areas in the western and northern BoB.

The simulation of climatological ISM rainfall is also well captured in the OML_OBS experiment, with a notable success in the simulation of rainfall over Central India. Due to the warm SST bias, there is an overall positive increase in rainfall over the oceans. However, the spatial structure of the rainfall is well captured; especially the climatologically strong rainfall over the Western Ghats and the northeastern BoB are realistically simulated (Fig. 1c).

**Figure 3:**
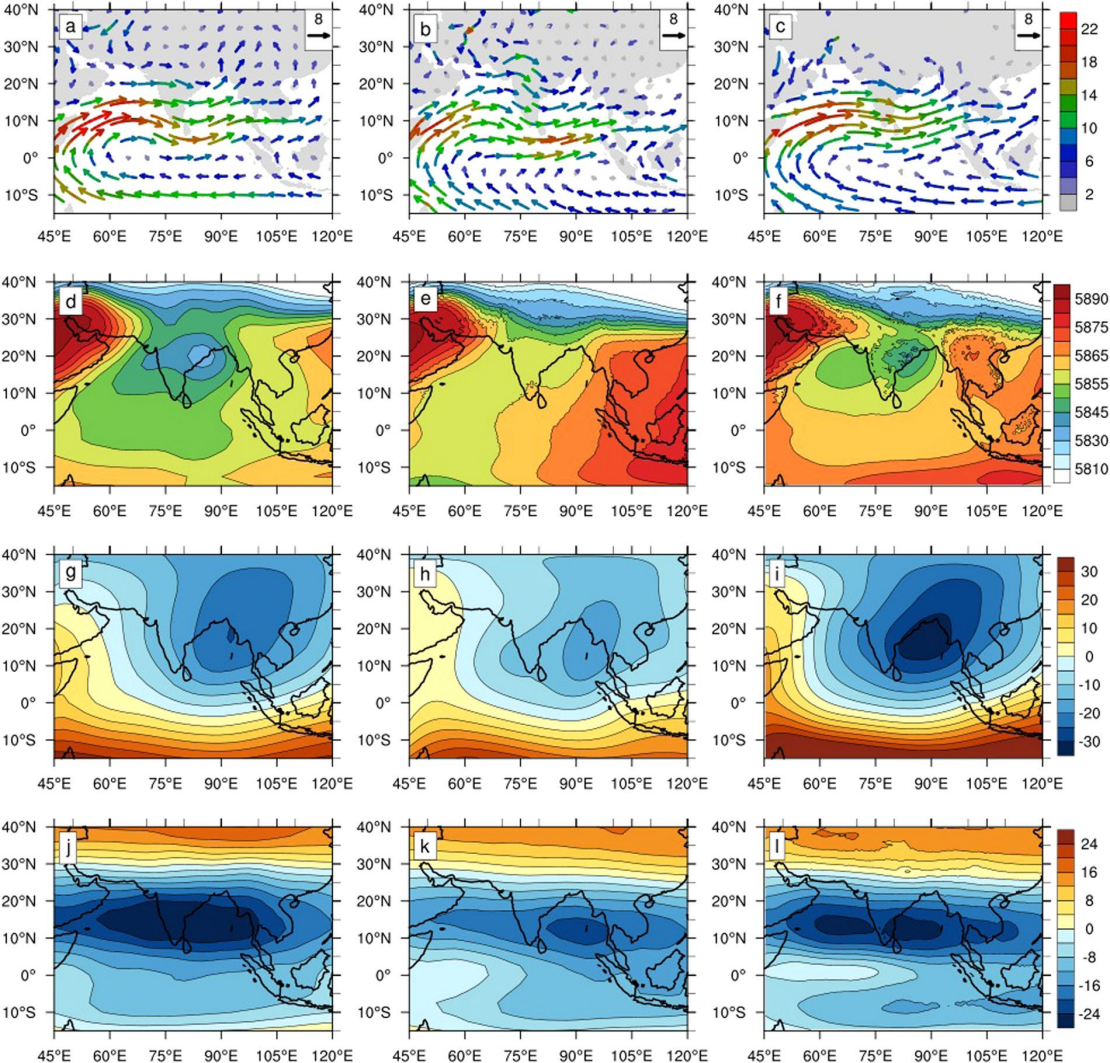
Spatial structure of seasonal climatological ISM state. The 850 hPa horizontal wind vector (unit: m/s; colors denote the wind speed) for (**a**) observations, (**b**) CFSv2, and (**c**) OML_OBS. The 500 hPa geopotential height (unit: m) for (**d**) observations, (**e**) CFSv2, and (**f**) OML_OBS. The 200 hPa velocity potential (unit: 10^6^ m^2^ s^−1^) for (**g**) observations, (**h**) CFSv2, and (**i**) OML_OBS. The 100 hPa zonal wind speed (unit: m/s) for (**j**) observations, (**k**) CFSv2, and (**l**) OML_OBS. Overall, vertical and horizontal wind shears are better captured in OML_OBS, better favouring the growth of monsoon depressions that affect Central India.

The OML_OBS experiment also improves upon the climatological state in CFSv2, producing more closer state to observations (Fig. 3). For example, the 850 hPa winds in CFSv2 (Fig. 3b) have a more diffuse and northward shifted Findlater jet, and a stronger anticyclonic vorticity over the Arabian Sea; further, the strongest winds at this level are shifted farther south than observations (Fig. 3a) over south India and the BoB. These biases are much less pronounced in the OML_OBS (Fig. 3c) run, although the low-level winds are shifted slightly to the north than in the observations. A remarkable improvement of ISM climate is the monsoon trough– the low-pressure region over the Indian landmass and northwestern BoB (Fig. 3d). While the monsoon trough is nearly absent at 500 hPa in CFSv2 (Fig. 3e), it is nearly as strong as the observations in OML_OBS (Fig. 3f). Related to the better simulation of rainfall patterns over the BoB and Central India, OML_OBS also simulates the upper tropospheric velocity potential and tropical easterly jet strength much better than CFSv2. Through these improvements, OML_OBS run offers a better representation of vertical and horizontal shears in the climatological ISM state. As a result, it likely provides an environment more conducive for the maintenance and growth of the LPS’s that are important for the rainfall over Central India^34,38,39^.

**Figure 4:**
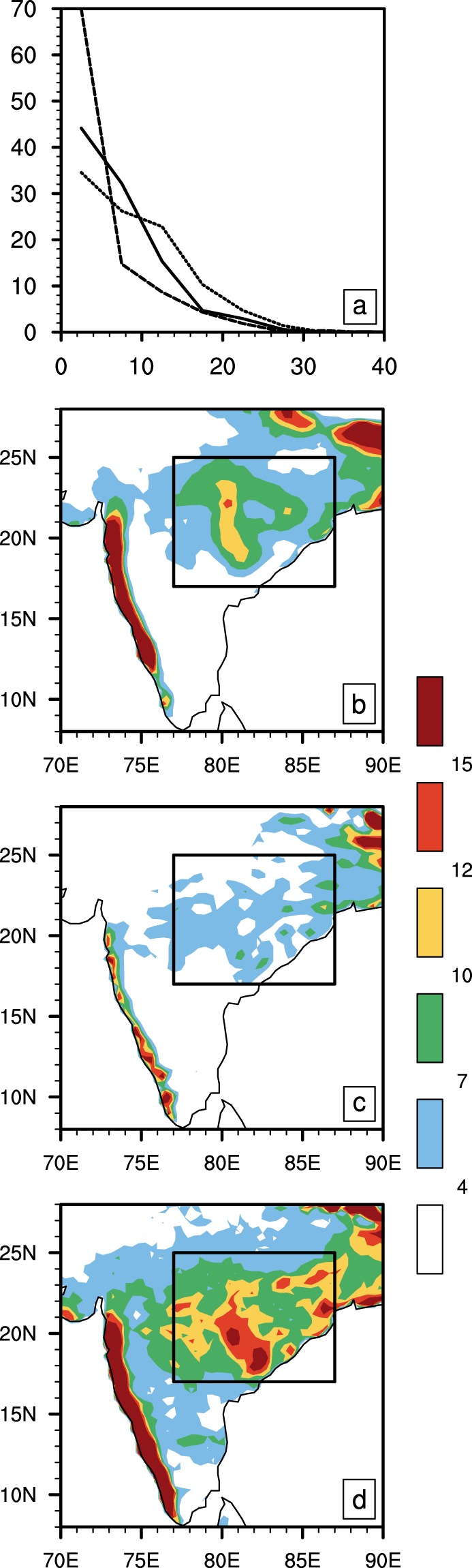
Histogram of daily mean rainfall and spatial distribution of heavy rainfall. (**a**) Histogram of daily rainfall over Central India (17–25°N, 77–87°E) for observations (solid line), CFSv2 (dotted line) and OML_OBS (dashed line). Spatial distribution of daily rainfall exceeding 25 mm/day for (**b**) observations, (**c**) CFSv2, and (**d**) OML_OBS. The X and Y-axis in the histogram denote bin center and probability density function (PDF, in %) respectively. The OML_OBS experiment captures the spatial distribution of heavy rain fairly well, while such events are concentrated to the east of the Central Indian domain in CFSv2 simulations.

Further, the OML_OBS run also simulates the intensity and spatial distribution of rainfall at synoptic scales better compared to CFSv2. Figure 4a shows the histogram of daily mean rainfall averaged over Central India (17–25°N, 77–87°E) from observations (solid line), CFSv2 (dotted line), and OML_OBS (dashed line). Consistent with previous studies^18^ most of the rain simulated by CFSv2 over Central India is from light rain events (less than 7 mm/day), with less frequent stronger rain events than observations. Though the OML_OBS experiment overestimates rain events stronger than 10 mm/day, the shape of the histogram is very similar to observations. The spatial distribution of regions experiencing greater than 25 mm/day rainfall in OML_OBS run (Fig. 4d) fairly matches with observations (Fig. 4b), whereas, CFSv2 exhibits stronger rainfall zones confined to the east of Central India domain, as discussed in *Srivastava et al*.^35^.

**Figure 5:**
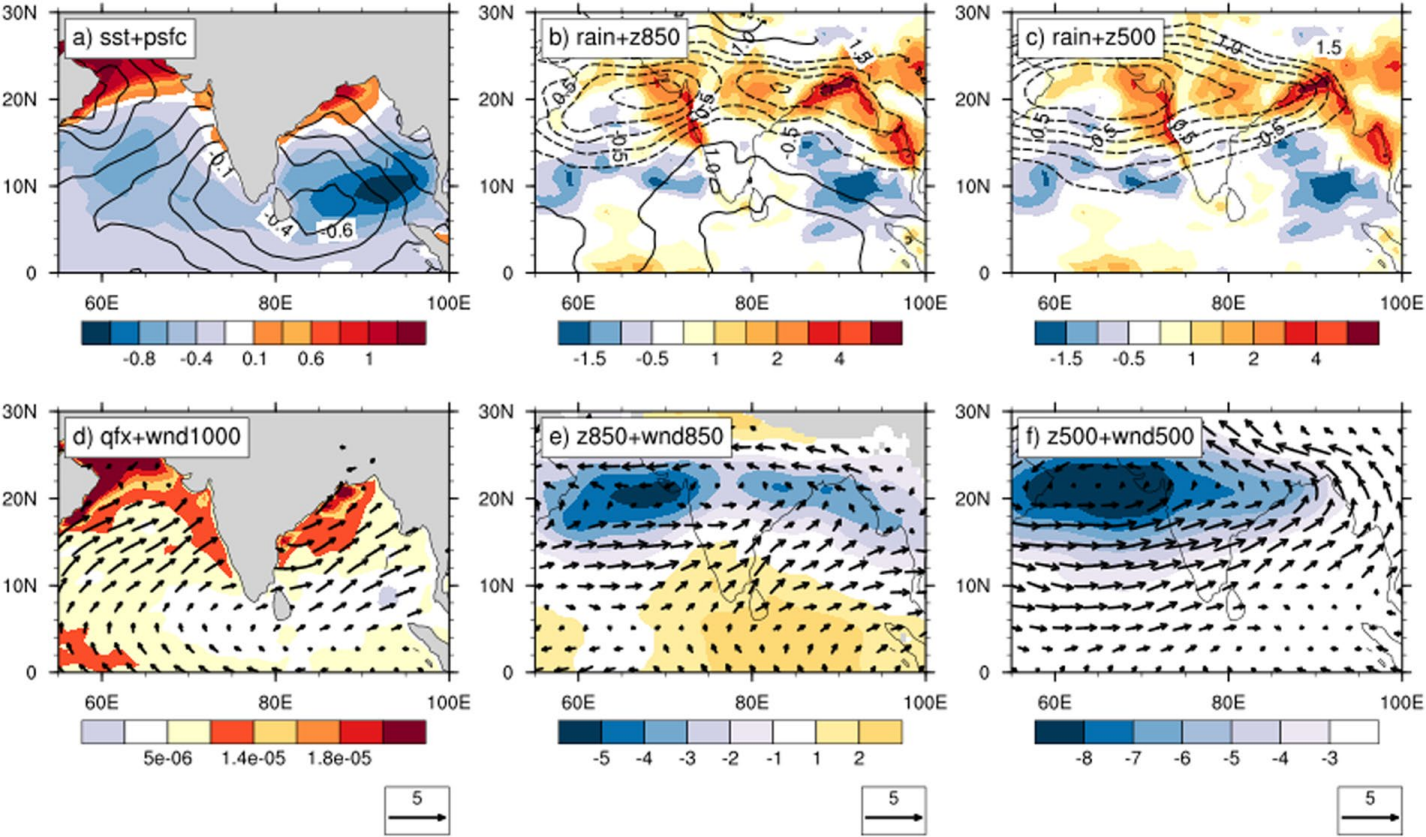
Sensitivity of ISM simulation to spatial variation of mixed layer depth (MLD). Differences between OML_OBS and OML_50M simulations for climatological state of (**a**) sea surface temperature (SST, shaded; unit: °C) and surface pressure (psfc, contour, unit: hPa), (**b**) rainfall (shaded, unit: mm/day) and 850 hPa geopotential height (z850, contour, unit: m), (**c**) rainfall (shaded) and 500 hPa geopotential height (z500), (**d**) 1000 hPa moisture flux (qflux, shaded; unit: kg.m^−2^ s^−1^ and 1000 hPa wind (vector, unit: m/s), (**e**) z850 and 850 hPa wind and, (**f**) z850 and 500 hPa wind.

What accounts for the remarkable simulation of mean ISM climate state by OML_OBS? It may be noted that, at large, the structure of CFSv2 biases resemble the differences between CFSv2 and OML_OBS. For example, CFSv2 simulates weaker rainfall over Central India compared to observations as well as OML_OBS, while its rainfall over southeast India is stronger compared to the latter two (Figs 3 and 5). Thus, OML_OBS appears to simulate multiple factors better, which are under-represented in CFSv2.

Interestingly, the differences between the OML experiments (OML_OBS-OML_50M) mimic CFSv2’s biases to a large extent (Figs 3 and 5)—the weaker rainfall over Central India in OML_50M compared to OML_OBS provides one example. Further, both CFSv2 and OML_50M simulate the monsoonal surface winds with their cores shifted southward relative to observations and OML_OBS (Figs 3 and 5). The monsoon trough is likewise underestimated in CFSv2 and OML_50M compared to the latter group. All these similarities suggest that a comparison between OML_50M and OML_OBS may provide valuable insights into the biases of ISM climatology present in CFSv2. Such a comparison is described next.

Figure 5 highlights the difference of OML_OBS from OML_50M in selected fields. The OML_OBS run simulates stronger rainfall not only over Central India but also over the northeastern BoB and northwest India. Further, its geopotential fields in the lower and middle troposphere are located at heights lower than, and are accompanied by stronger cyclonic circulations than, that of the OML_50M run. However, at the surface, OML_OBS has higher surface pressure, with the largest differences east of Sri Lanka. The surface wind differences appear to be a result of both the stronger cyclonic tendency at the mid-tropospheric levels and the stronger anticyclonic tendency at the surface—in contrast to the wind differences at the 850 and 500 hPa levels which are clearly cyclonic. As expected, warmer SSTs are simulated in OML_OBS over the western BoB. Overlying the warmer SST, surface moisture fluxes are higher and surface pressure is lower while surface winds are cyclonic in OML_OBS relative to OML_50M.

The differences between the OML_OBS and OML_50M runs may be linked to the differences in their simulation of climatological SST patterns. As the MLD structure is the only difference in the set up of the experiments, the shallow mixed layer in the OML_OBS experiment allows it to reproduce the narrow coastal SST front in the northwest BoB reasonably close to observations. Consequently, enhanced surface heating and latent heat fluxes introduce a narrow low-pressure front (Fig. 5a) lying atop the SST front, which enhances the monsoonal winds overlying the front. Development of deep convection (Fig. 5b) in warmer SST regions elsewhere in the OML_OBS run also help enhance and extend the winds across the northern BoB. The enhanced winds impinge on the steep topography at the north-eastern coast of the BoB and the resulting uplift produces enhanced convection, which further strengthens the monsoonal winds across the BoB. The enhanced mid-tropospheric lows over the northern BoB may be related to the enhanced rainfall over this region in OML_OBS. Meanwhile, the enhanced winds cool SST through surface fluxes, with more stronger cooling under the core of the mean winds (due to non-linear effects), which are found around 10°N. The enhanced surface pressure lying east of Sri Lanka may be related to the cooler SST simulated in OML_OBS over this region, relative to OML_50M.

However, the enhanced rainfall over Central India cannot be readily explained by the structure of the wind differences between the two runs. While local rainfall overlying the coastal SST front can be explained due to the combined effects of surface heating and fluxes, and eastern BoB rainfall explained in terms of circulation-orography interaction, the pattern of wind differences do not suggest enhanced moisture transport directed inland towards Central India from the BoB. However, meridional and vertical wind shears are both enhanced in the OML_OBS compared to OML_50M run. This is likely to increase the growth (both frequency and intensity) of LPS over Central India, enhancing rainfall over the region.

**Figure 6:**
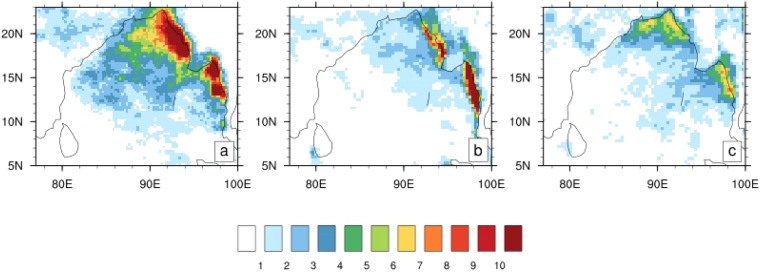
Spatial distribution of extremely strong rainfall in the BoB. The seasonal climatological pattern of extremely strong rainfall (more than 60 mm/day) for (**a**) observations, (**b**) CFSv2, and (**c**) OML_OBS. In observations, strong rainfall has a noticeable northwest–southeast orientation. This structure of rainfall is well reproduced in both the simulations. A secondary feature in the observations is the zone of rainfall oriented southwest–northeast, parallel to the eastern coastlines of India. This feature is not captured by CFSv2, which has a marked lack of strong rainfall over the western BoB. The OML_OBS simulation captures this feature relatively better, likely due to the reasonable simulation of the warm SST front underlying this region.

We posit that the warm SST front in the northwestern BoB increases the probability for these systems to penetrate into and strengthen over Central India by favouring the formation of genesis vortices closer to the coastlines adjoining Central India. The distribution of extremely strong rainfall (heavier than 60 mm/day) over the BoB (Fig. 6) provides some support for this hypothesis. It is interesting that the observed pattern of strong rain over the BoB not only has a southeast to northwest orientation but also oriented parallel to both the northeast coastline of India and the narrow coastal BoB SST front. In observations, this zone becomes more prominent as stronger rainfall events (implying deeper, intense updrafts) are considered (Fig. S4). The deep and strong updrafts in these rainfall zones can generate deep vortex cores through convergence/stretching and possibly vertical advection of absolute vorticity^40^, which in turn can form the seeds of LPS systems. This zone is prominent in both observations (Fig. 6a) and OML_OBS run (Fig. 6c), but absent in CFSv2 (Fig. 6b). The deep convection in CFSv2 is confined to the northeastern BoB. This is consistent with the earlier study of *Srivastava et al*.^35^ that shows the most LPS in the CFSv2 form in the northeastern or central BoB, and likely weaken in intensity before reaching Central India. This may explain why intense rainfall over land is confined to the coastline close to the north BoB (Fig. 5c), as well as the dry bias over Central India in CFSv2.

**Figure 7:**
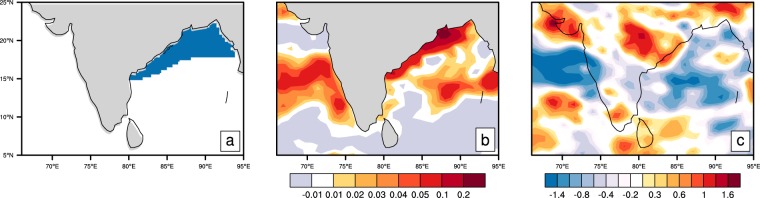
A sensitivity experiment demonstrating the role of mixed layer depth (MLD) on SST and rainfall in the tropical Indian Ocean. (**a**) The shaded area shows the region where MLD was artificially deepened by 10 m in a perturbed MLD experiment. The mixed layer was deepened only in regions where the climatological (June–September) depths were equal to or less than 12 m. (**b**) Show differences of SST climatology (unit: °C) in the climatological MLD runs from that of the perturbed MLD runs. (**c**) Is the same as (**b**), but shows differences of rainfall climatology (unit: mm/day) between the runs.

We have carried out a final set of experiments to isolate the role of the shallow BoB MLD region for the warm SST front, and consequently for rainfall over central India. In the first experiment, the WRF model was coupled to the ocean mixed layer model with climatological (June–September) time-invariant MLD. The lateral boundary conditions for these runs were provided from CFSv2 for the seven years from 1999–2005. The second experiment was similar to the first, except that the MLD was artificially deepened by 10 m in regions where observed MLD was shallower than 12 m (Fig. 7a).

In Fig. 7b, we show the climatological differences for SST between the experiments. Positive values indicate that SST is warmer in the first experiment compared to the second. It is clear that the shallower mixed layer over the northwestern BoB helps promote the warm SST front in that region. Note that significant SST differences are also noted elsewhere in the Indian Ocean, outside the area where MLD was perturbed. In general, they seem to be related to the differences in climatological rainfall (Fig. 7c) between the experiments: warm SST differences in regions with diminished rainfall, and vice versa. The rainfall differences (Fig. 7c) show local enhancement of rainfall over the warm SST front in the BoB, and over the adjacent central Indian region highlighted in this study. This excessive zone of rainfall is seen extending northwestwards over Central India, and extends further along the axis of the climatological monsoon trough regions. Overall, all the experiments shown here suggest that the structure of the oceanic mixed layer in the BoB is a key factor that influences climatological aspects of ISM rainfall. Further experiments and analyses are needed to understand the role of the oceanic mixed layer in variability of the monsoon rainfall at intraseasonal and longer timescales.

## Discussion

Our findings provide fresh insights into the issue of ISM simulations, especially its climatological biases in CGCMs. The climatological bias is mostly discussed in the literature^2,17,36^ in terms of rainfall over Central India. However, the bias is in fact more systemic and involves biases in important circulation features such as the monsoon trough. Thus, our findings have far-reaching implications beyond the dry bias over Central India.

As far as we are aware, the warm SST front over the BoB, highlighted in this study has not been discussed elsewhere, although the features of the mixed layer that gives rise to the front in the BoB are discussed in *Shenoi et al*.^26^. This finding itself is consequent to the availability of satellite microwave observations which can measure SST in the presence of clouds. It is well known that clouds are frequently present in the BoB during the ISM season, and that infrared techniques cannot observe SST through clouds; the SST front is also present in infrared satellite observations, but with the sharp gradients of the front somewhat smoothed out. Further, while the northwest–southeast orientation of BoB convective maxima is well discussed in the literature^19^, the secondary maxima oriented along the western BoB coastline is not properly addressed before. This finding also owes itself to the availability of direct measurements of precipitation from the Tropical Rainfall Measuring (TRMM) Precipitation Radar (PR). The implications of this infrequent, but intense and deep convection zone parallel to the SST front, for monsoon air-sea interaction and LPS genesis are profound.

We identify two factors that may be responsible for the improvement of Central Indian rainfall in the OML_OBS experiment. The better simulation of horizontal and vertical shears in this run favours the enhanced growth of LPS, thereby enhancing rainfall over Central India. However, it is likely that the circulation changes associated with the increased horizontal and vertical shears are a consequence of the enhanced rainfall and not vice versa. It is more likely that the enhanced Central Indian rainfall in the OML_OBS experiment is a consequence of its LPS genesis forming closer to the western BoB coastline. This, in turn, can be related to better simulation of the coastal SST front in the BoB in this experiment, which breeds deep and intense convective updrafts in its vicinity. Conversely, the poor simulation of CFSv2 in this respect may be related to its poor simulation of the SST front. By focusing on this aspect, and its relation to oceanic mixed layer structure in the CFSv2 and other CGCM simulations, our work offers a new way forward in understanding and rectifying the ISM rainfall bias in climate simulations.

## Observation, Model, and Methods

We used merged TMI-Aphrodite daily rainfall data is using Aphrodite daily rainfall products^41^ and TRMM 3B42 v7 daily products^42^, as Aphrodite data was available only over the land. We denote this merged product as observational rainfall throughout, unless otherwise mentioned. The domain of Central India for this study is considered over 17°N–25°N, 77°E–87°E. The MLD data (Fig. 2a and experiments) is based on observed subsurface ocean temperature and salinity profiles available from https://www.metoffice.gov.uk/hadobs/en3/. Observational SST is used from monthly TMI products available from http://www.remss.com/missions/tmi. Extreme rainfall analysis (Figs 6 and S4) is done using daily TRMM PR 2A25 version 7 products^43^ during 1999–2005. Geopotential height and wind products are used from NCEP-NCAR reanalysis^44^ products. All the plots shown here are seasonally averaged over June-July-August-September (JJAS).

### CFSv2 model

The CFSv2^11^ is a fully coupled ocean-atmosphere-land-sea ice model with advanced physics, with increased resolution and refined coupled initialization in comparison to earlier version CFSv1. The CFSv2 version is similar to the version of NCEP model used for climate forecast system reanalysis CFSR^45^. The atmospheric component of CFSv2 is the NCEP Global Forecast System (GFS) model, configured with a spectral triangular truncation of 126 waves (T126) in the horizontal (0.9° grid) and 64 sigma-pressure hybrid levels along vertical using finite differencing. Whereas, the ocean component is Geophysical Fluid Dynamics Laboratory Modular Ocean Model version 4p0d (MOM4p0d)^46^ with the finite difference of ocean primitive equations, configured under horizontal grid spacing under Boussinesq and hydrostatic approximation. The zonal resolution is 0.5° and meridional resolution is 0.25° within 10°S and 10°N. The meridional resolution becomes gradually coarser through the tropics and up to 0.5° poleward of 30°S and 30°N. The MOM4p0d contains 40 vertical layers with 27 in the upper 400 m, along with an approximate bottom depth of 4.5 km. Up to 240 m depth from the surface, the vertical resolution is 10 m and then gradually increases to about 511 m in the bottom layer^47^. The atmosphere and ocean models are coupled with no flux adjustment or correlation and use simplified Arakawa-Schubert convection with momentum mixing. The model implements orographic gravity wave drag^48^ and sub-grid scale mountain blocking by *Lot and Miller*^49^. The model is coupled with a four-layer Noah land surface model^50^ and a two-layer sea ice model^51^. The CFSv2 free runs of 30 years are performed on Prithvi HPC system at Indian Institute of Tropical Meteorology, Pune. The atmospheric initial conditions for the coupled free runs are based on reanalysis of *Kanamitsu et al*.^52^ data and the ocean initial conditions are stand on NCEP Global Ocean Data Assimilation System (GODAS). We used CFSv2 free run for 7 years (1999–2005) for this study at T382 resolution.

### WRF model

We used the Advanced Research WRF (ARW) version 3.4.0, which is an atmosphere-only, limited area, mesoscale modeling system and has been widely used for atmospheric research. It solves a set of fully compressible, non-hydrostatic and Eulerian equations in terrain-following coordinates^53^. The model physics used in this study are summarized in Table 1. The same set of model physics has been used by several studies for regional rainfall study. WRF is also widely used for ISM study^54^. We chose a single domain (Fig. S2) for our study over 28°S–38°N, 40°E–135°E (black box) with 214 (south-north) × 271 (west-east) grid points. The configuration consists of 38 km horizontal resolution and a coupling with slab ocean mixed layer model. The model top is fixed at 50 hPa. The daily model outputs from WRF are saved for further analysis. The wide range of elevation over the Indian subcontinent in Fig. S2 shows the orographic complexity over the region. Understanding regional processes due to topography in tropical regions have a crucial role in monsoonal rainfall^19,55^.

**Table 1:** Physical schemes used for the WRF simulations.

Model Physics	Physical Schemes Used
Cumulus	Betts-Miller-Janiac^60^
Longwave radiation	RRTMG^61^
Shortwave radiation	RRTMG^61^
Land surface	Unified Noah land surface model^50^
Surface layer	Monin-Obukhob (Janiac Eta)^60^
Boundary layer	Mellor-Yamada (Eta) TKE^61^
Microphysics	WSM 3-class simple ice^62^

### Coupled modeling experiments

To understand the impact of coupled ocean-atmosphere interactions, we conducted experiments coupling WRF with a one-dimensional ocean mixed layer model^56^. In one experiment, hereafter referred to as OML_OBS, the ocean model was initialized using the observed MLD climatology for June–September. In another experiment, hereafter OML_50M, the ocean model was initialized using a spatially uniform depth of 50 m. A final sensitivity experiment was conducted to lend support to our contention that the narrow region of warm SST had a significant effect on central Indian rainfall. For this experiment, the observed climatological MLD was artificially deepened wherever it was 12 m or shallower. The lateral boundary conditions for all the WRF runs were the same, and were obtained from daily resolution CFSv2 freerun data for 1999–2005.

### Track analysis

We used ERA-interim^57^ 6-hourly 850 hPa relative vorticity data at 0.5° horizontal resolution for 1979–2015 to identify monsoon low-pressure systems. Composite horizontal structure of low-pressure systems is obtained using NCEP-NCAR reanalysis^44^ daily field of mean sea level pressure and surface winds (0.995 sigma level). An automated tracking algorithm, the TRACK programme^58,59^ is used to identify feature tracks. We applied TRACK to 850 hPa relative vorticity for 1979–2015. The program is used to identify relative vorticity maxima above a threshold amplitude of 1.0 × 10^−5^ s^−1^, and locate the feature in time and spherical latitude-longitude. After tracing relative vorticity maximum, the programme works by minimizing a cost function for the speed and direction of motion in 6-hourly reanalysis^58^ output. Outputs from TRACK program is filtered further by following *Hurley and Boos*^39^. We identified monsoon lows, monsoon depression, and cyclonic storms separately. The monsoon depression is the major contributor for ISM rainfall, therefore shown in Fig. 2a. In the text, we mean both lows and monsoon depression by referring to LPS.

## Electronic supplementary material

Supplementary Information

## Data Availability

Since the model source code is not open access and model simulations are run on the experimental basis, the model data is not available publicly as of now. The output from the WRF experiments can be provided on request. All the observational data sets are available on respective sites as mentioned in the manuscript.
