# Supplementary material for: Impact of a Narrow Coastal Bay of Bengal Sea Surface Temperature Front on an Indian Summer Monsoon Simulation
Source: Sci Rep. 2018 Dec 6;8:17694. doi: 10.1038/s41598-018-35735-3 (PMC6283853; doi:10.1038/s41598-018-35735-3)
Supplement: Supplementary file 1 — Supplementary Information [file 41598_2018_35735_MOESM1_ESM.docx]

**SUPPLEMENTARY FILE**

Impact of a narrow coastal Bay of Bengal sea surface temperature front on an Indian summer monsoon simulation

Dhrubajyoti Samanta^1,2^, Saji N. Hameed^1,*^, Dachao Jin^1,3^, Vishnu Thilakan^1^, Malay Ganai^4^, Suryachandra A. Rao^4^ and Medha Deshpande^4^

1. Environmental Informatics, University of Aizu, Japan

2. Present address: Asian School of the Environment, Nanyang Technological University, Singapore

3. Present address: Collaborative Innovation Center on Forecast and Evaluation of Meteorological Disasters, Nanjing University of Information Science and Technology, China

4. Indian Institute of Tropical Meteorology, India

* Corresponding author: Saji N. Hameed, Email: saji@u-aizu.ac.jp

**Figure legends**

1. Figure S1: **Seasonal (JJAS) climatological rainfall bias** (model-observations; unit: mm/day). (a) CFSv2, (b) OML_OBS, and (c) OML _50m (unit: mm/day). The severe bias of CFSv2 over central India and north-western BOB is reduced in OML_OBS experiment.

2. Figure S2: **WRF model domain marked with the red box.** The shaded values show the elevation (unit: m) from ETOPO5 data, available from Data Announcement 88-MGG-02, Digital relief of the Surface of the Earth. NOAA, National Geophysical Data Center, Boulder, Colorado, 1988.

3. Figure S3: **Sanity checks for OML_OBS experiment using 500 hPa geopotential height.** The line plots are averaged over 0-30 ºN and 40-110 ºE for NCEP, CFSv2, and OML_OBS (unit: m). The seasonal climatological plots are for March-April-May (MAM), June-July-August (JJA), September-October-November (SON) and December-January-February (DJF) seasons. The X-axis denotes the number of days in the season. The OML_OBS experiment simulates the time evolution of 500 hPa geopotential reasonably well.

4. Figure S4: **Spatial distribution of extremely strong rainfall from TRMM PR observations.** Seasonal climatological pattern of number of extremely strong rainfall events for (a) 60 mm/day, (b) 80 mm/day, (c) 100 mm/day, and (d) 120 mm/day. Stronger the convection, more likely it stays near the SST front in the western BoB, thereby increasing the probability of genesis vortices to penetrate into Central India.


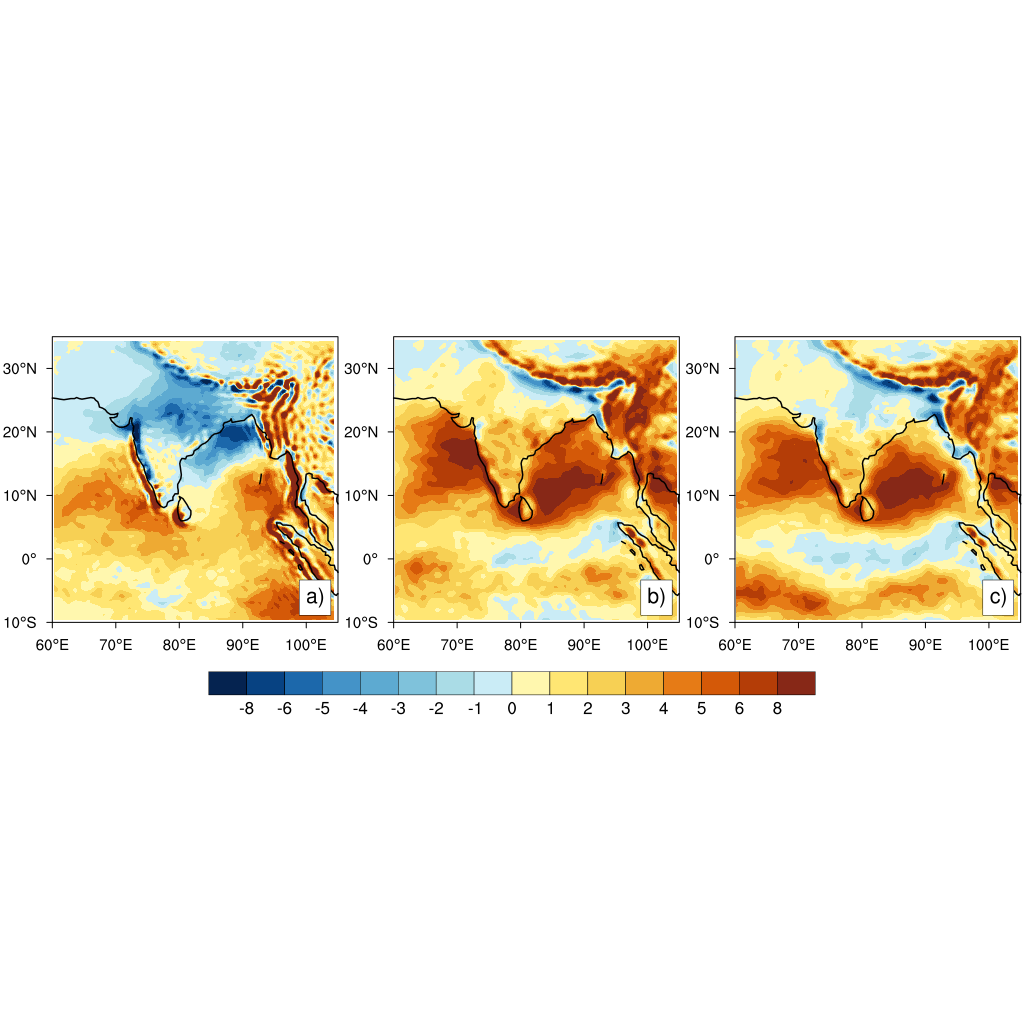


Figure S1: **Seasonal (JJAS) climatological rainfall bias** (model-observations; unit: mm/day). (a) CFSv2, (b) OML_OBS, and (c) OML _50m (unit: mm/day). The severe bias of CFSv2 over central India and north-western BOB is reduced in OML_OBS experiment.


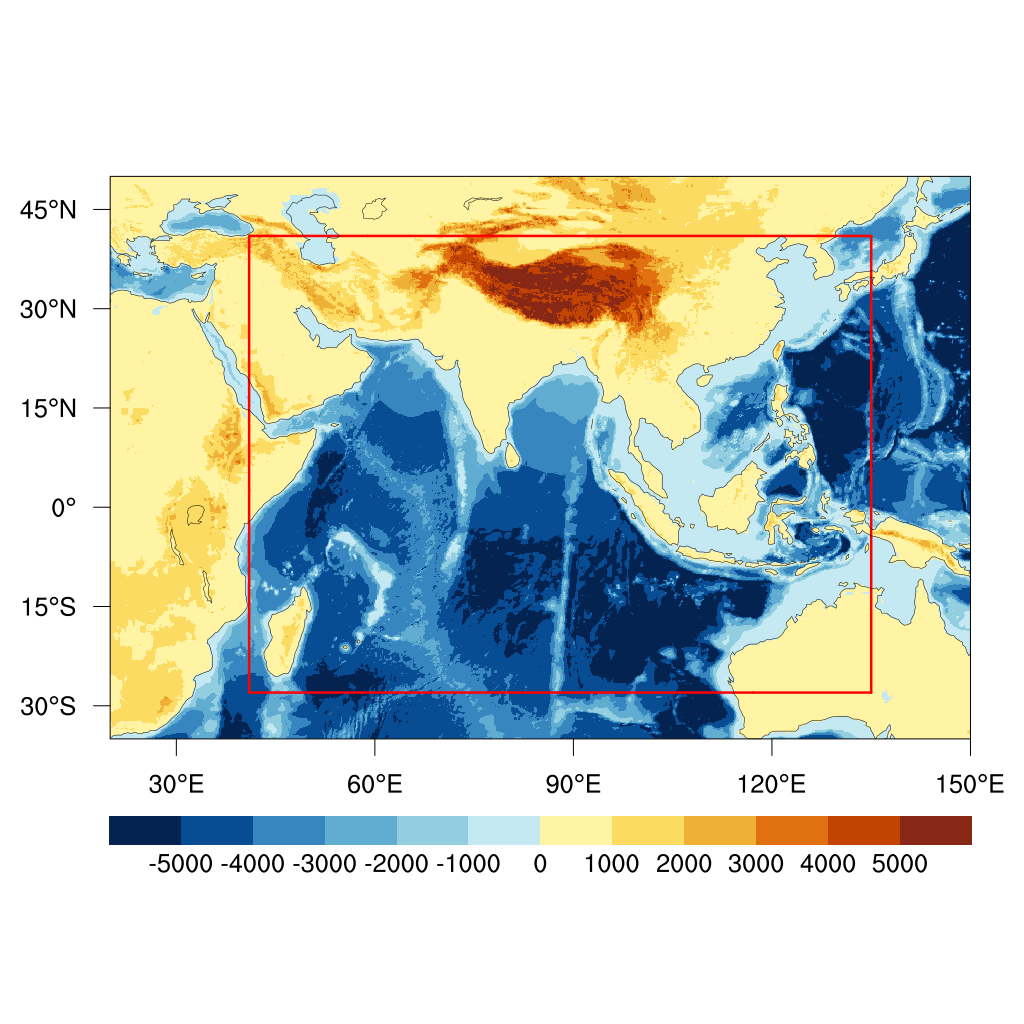


Figure S2: **WRF model domain marked with the red box.** The shaded values show the elevation (unit: m) from ETOPO5 data, available from Data Announcement 88-MGG-02, Digital relief of the Surface of the Earth. NOAA, National Geophysical Data Center, Boulder, Colorado, 1988.


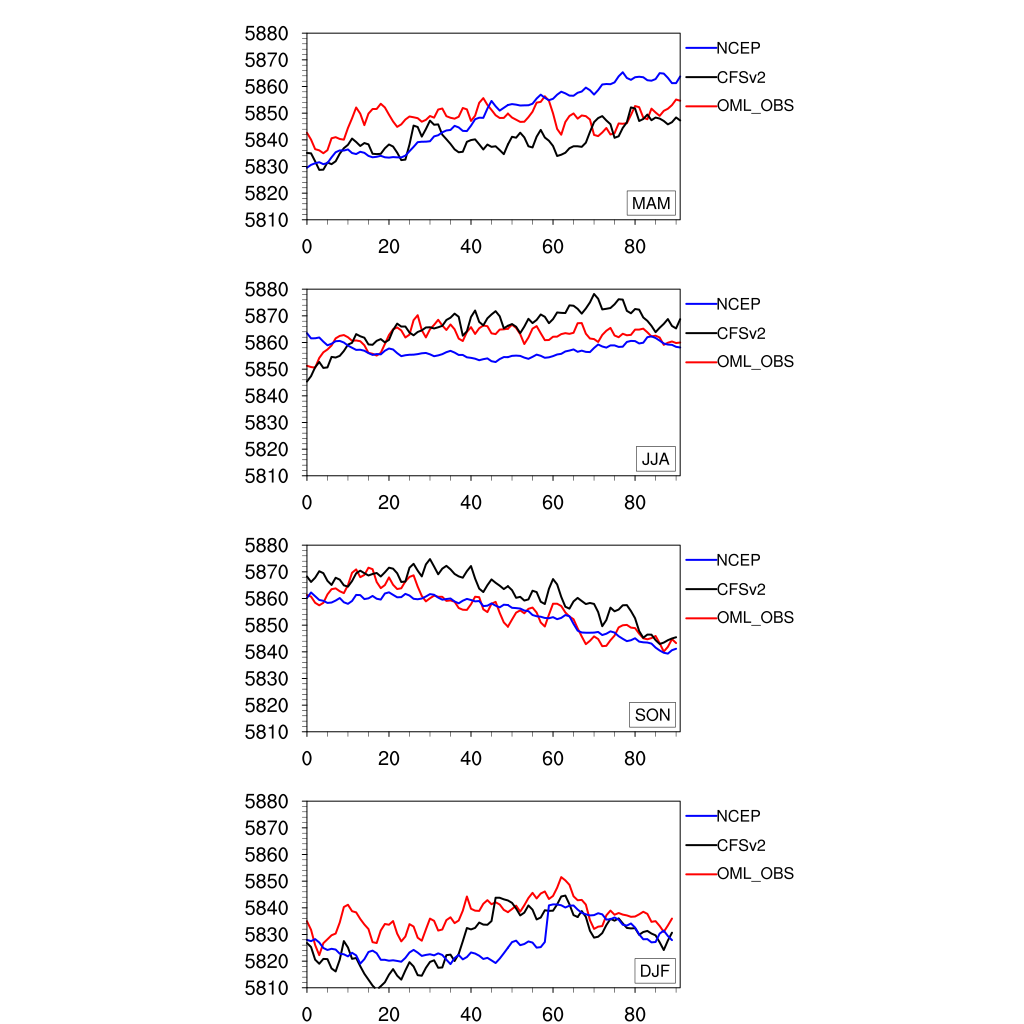


Figure S3: **Sanity checks for OML_OBS experiment using 500 hPa geopotential height.** The line plots are averaged over 0-30 ºN and 40-110 ºE for NCEP, CFSv2, and OML_OBS (unit: m). The seasonal climatological plots are for March-April-May (MAM), June-July-August (JJA), September-October-November (SON) and December-January-February (DJF) seasons. The X-axis denotes the number of days in the season. The OML_OBS experiment simulates the time evolution of 500 hPa geopotential reasonably well.


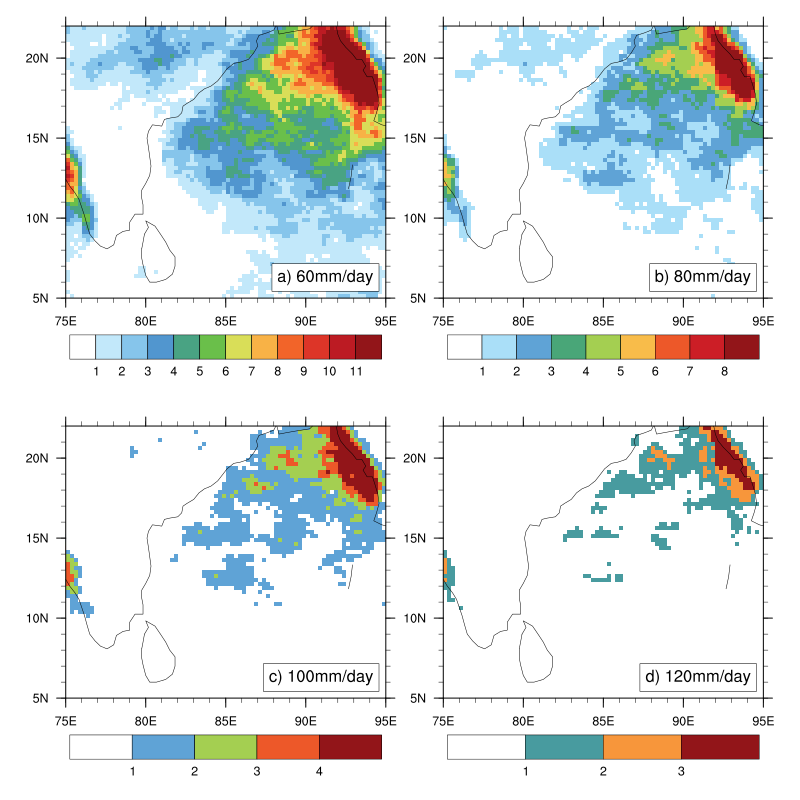


Figure S4: **Spatial distribution of extremely strong rainfall from TRMM PR observations.** Seasonal climatological pattern of number of extremely strong rainfall events for (a) 60 mm/day, (b) 80 mm/day, (c) 100 mm/day, and (d) 120 mm/day. Stronger the convection, more likely it stays near the SST front in the western BoB, thereby increasing the probability of genesis vortices to penetrate into Central India.
